# Cancer and COVID-19: analysis of patient outcomes

**DOI:** 10.2217/fon-2021-0121

**Published:** 2021-07-15

**Authors:** Mohamed Aboueshia, Mohammad Hosny Hussein, Abdallah S Attia, Aubrey Swinford, Peter Miller, Mahmoud Omar, Eman Ali Toraih, Nakhle Saba, Hana Safah, Juan Duchesne, Emad Kandil

**Affiliations:** ^1^Department of Surgery, Division of Endocrine & Oncologic Surgery, Tulane University, School of Medicine, New Orleans, LA 70112, USA; ^2^Department of Histology & Cell Biology, Genetics Unit, Faculty of Medicine, Suez Canal University, Ismailia, 41522, Egypt; ^3^Tulane University, School of Medicine, New Orleans, LA 70112, USA; ^4^Trauma/Acute Care & Critical Care, Department of Surgery, Tulane, Tulane School of Medicine, New Orleans, LA 70112, USA; ^5^Section of Hematology & Medical Oncology, Deming Department of Medicine, Tulane University, New Orleans, LA 70112 USA; ^6^Department of Otolaryngology-Head & Neck Surgery, Faculty of Medicine, Suez Canal University, Ismailia, 41522, Egypt; ^7^Department of Surgery, Tulane University School of Medicine, New Orleans, LA 70112 USA

**Keywords:** active cancer and remission, breast cancer, cancer, comorbidities, COVID-19, delayed management, lung cancer, outcomes, prostate cancer

## Abstract

**Background:** We sought to investigate the outcomes associated with COVID-19 disease in cancer patients. **Methods:** We conducted a retrospective cohort study of laboratory-confirmed COVID-19 patients. **Results:** Of the 206 patients included, 57 had at least one preexisting malignancy. Cancer patients were older than noncancer patients. Of the 185 discharged cases, cancer patients had a significantly higher frequency of unplanned reintubation (7.1% vs 0.9%, p < 0.049), and required longer hospital stay (8.58 ± 6.50 days versus 12.83 ± 11.44 days, p < 0.002). Regression analysis revealed that obesity and active smoking were associated with an increased risk of mortality. **Conclusion:** Outcomes in COVID-19 appear to be driven by obesity as well as active smoking, with no difference in mortality between cancer and noncancer patients.

## Summary

In this study, we aimed to investigate how COVID-19 affected cancer patients and whether this altered their survival outcomes. To do this, we examined data from a database of patients who have passed through our institution – a retrospective cohort analysis. Of the 206 patients we included in the study from this database, 57 had at least one preexisting cancer. Cancer patients tended to be older than noncancer patients. Of the 185 discharged patients, cancer patients required longer hospital stays, but there was no difference in mortality. Disease complications and intensive care unit admission with obesity and active smoking put patients in our cohort at increased risk of death. To conclude, outcomes in COVID-19 patients appear to be driven by obesity as well as active smoking, with no difference in mortality between cancer and noncancer patients.

As the COVID-19 epidemic evolves, countries are considering policies of shielding putative vulnerable patients at increased risk of severe phenotype. A consistently high risk of severe COVID-19 and adverse outcomes in older individuals and those with underlying health conditions has been shown. The CDC has issued guidelines on persons at increased risk of severe COVID-19 that include people with cardiovascular disease, diabetes, chronic renal disease and a range of other chronic conditions [1]. However, information about the risk of severe disease among many comorbidities is scarce. Currently, limited literature exists on the risk of severe illness from COVID-19 in cancer patients relative to patients without cancer.

The COVID-19 pandemic has forced clinicians to choose between providing pre-COVID-19 standards of care and altering care to reduce exposure of at-risk patients to COVID-19. Older individuals and those with underlying conditions have consistently been shown to have a higher risk of severe COVID-19 illness and adverse outcomes [2]. This gives rise to the ethical dilemma physicians face, especially with the shortage in resources the health system has been exposed to during the pandemic and the scarcity of data available in the literature on how to manage these patients [3,4]. Another ethical consideration that should be taken into account is the stress the cancer patients experience if their treatment was postponed [5].

Early evidence from China suggested increased risk of COVID-19 and adverse events in cancer patients; however, the evidence supporting these conclusions was lacking [6,7]. Later meta-analysis of studies from China and Italy showed an increased risk for COVID-19 and adverse events in cancer patients compared with the general population, although the study authors acknowledged that experts should only cautiously extrapolate from the study results [8]. Studies in the USA related to COVID-19 and cancer are ongoing, but data from the New York State Department of Health shows that as of 14 August 2020, 7.6% of deaths attributable to COVID-19 were in patients with cancer [9]. Cumulatively, these data suggest that cancer patients are at greater risk for mortality and adverse outcomes from COVID-19, but interpretation should be made cautiously. Epidemiological data within the US remain limited, and cancer patients are a diverse cohort.

This article attempts to study the relationship between cancer and severe COVID-19 illness with adverse outcomes. We hope this will contribute to the literature available to help physicians better manage the treatment of cancer patients during the pandemic.

## Methods

### Study population

Study data were collected and managed using REDCap electronic data capture tools hosted at Tulane Medical Center. REDCap (Research Electronic Data Capture) is a secure, web-based software platform designed to support data capture for research studies, providing an intuitive interface for validated data capture and audit trails for tracking data manipulation and export procedures [10]. Adult hospitalized patients were collected during the period between 27 February and 27 April 2020. COVID-19 testing for patients was done using RT-PCR through the Louisiana Office of Public Health (OPH) Laboratory. Institutional review board (IRB) approval was obtained. Cancer patients were divided into patients receiving treatment as active cancer and patients in remission with nonactive cancer.

### Variables

Demographics, clinical presentation, comorbidities, laboratory values on admission, complications and outcomes were collected. PaO_2_/FIO_2_ ratio was estimated as the ratio of arterial oxygen partial pressure (PaO_2_ in mm Hg) to fractional inspired oxygen [11]. The neutrophil–lymphocyte ratio was determined an indicator for poor outcome [12]. Two scoring systems were used for severity assessment: first, the CURB-65 score – confusion status, blood urea nitrogen level >19 mg/dl (>7 mmol/l), respiratory rate ≥30, blood pressure (systolic <90 mm Hg or diastolic ≤60 mm Hg) and age ≥65 years [13]; second, the Quick Sequential Organ Failure Assessment (qSOFA) score based on Glasgow Coma Score <15, respiratory rate ≥22 and systolic blood pressure ≤100 [14].

### Outcomes

Comparisons between cancer and noncancer groups and between active and nonactive cancer cohorts were performed. The primary outcome of concern was in-hospital mortality. Secondary outcomes were risk of intensive care unit (ICU) admission, risk of endotracheal intubation, duration of mechanical ventilation and length of hospital stay.

### Statistical analysis

Data management was carried out using SAS 9.4 (SAS Institute Inc., 2013, NC, USA), and statistical analysis was performed using STATA 16.0 (StataCorp., 2019, TX, USA) and SPSS 26.0 (SPSS, Inc., IL, USA). Quantitative data were described as mean and standard deviation (SD), and binary variables were presented as frequencies and percentages. Chi-square or Fisher's exact tests were applied to test the differences in categorical variables, and Student's t- and Mann-Whitney U-tests were used for quantitative variables to examine the difference between cancer and noncancer patients and between active and nonactive cancer patients. Kaplan-Meier survival curves were plotted, and the log rank test was used to compare in-hospital mortality between groups. Binary logistic regression analysis was used to evaluate predictors of ICU admission and mortality in COVID-19 patients. Results were reported as odds ratio (OR) and 95% CI and two-sided p-values <0.05 were set as significant.

## Results

### Characteristics of the study population

**Figure 1.**
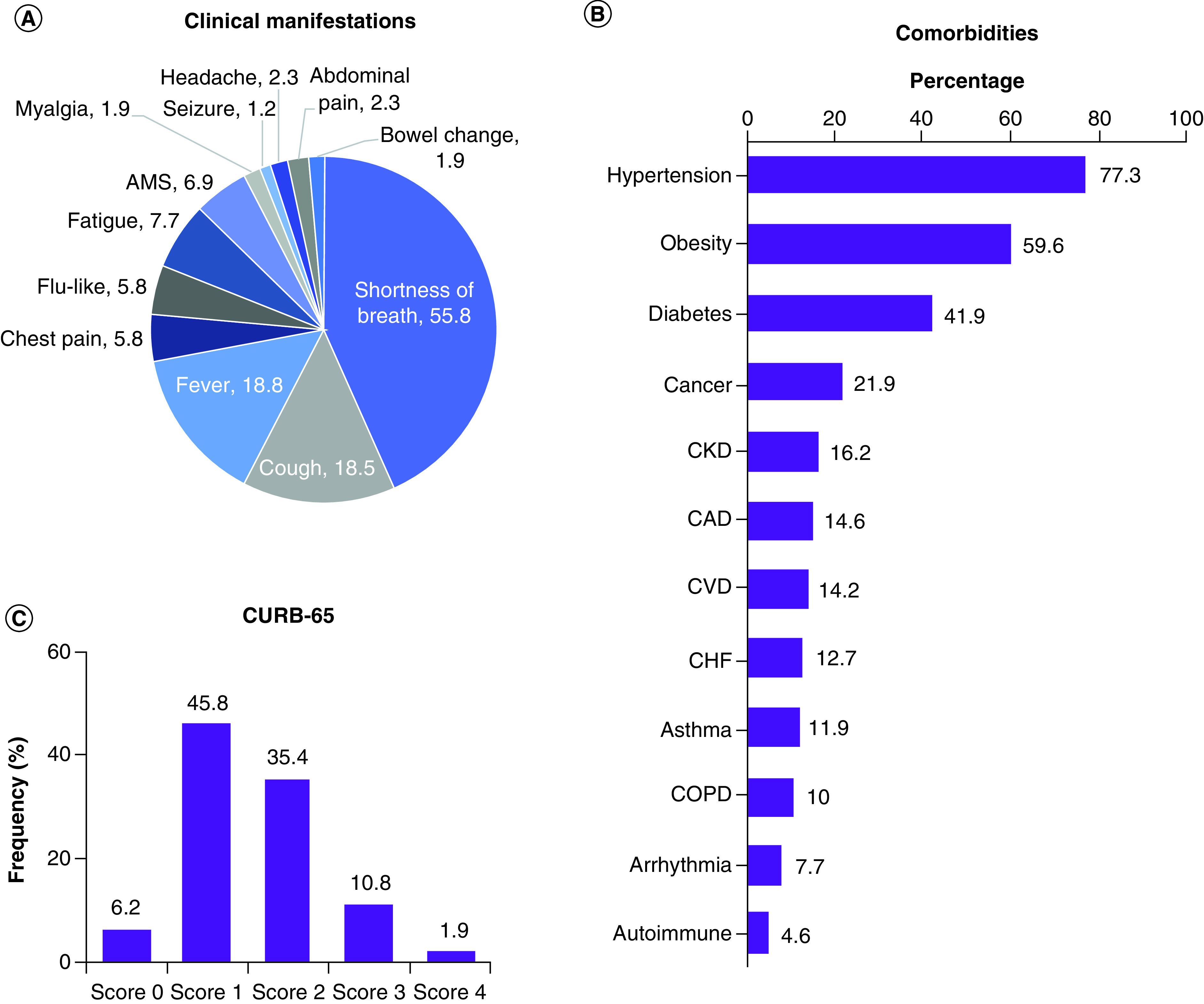
Clinical assessment of COVID-19 patients. **(A)** Clinical presentation on admission. **(B)** Frequency of comorbidities. **(C)** Severity assessment by CURB-65 score. CURB-65 score represents confusion (mental test score ≤8 new disorientation in person, place or time), blood urea nitrogen >20 mg/dl, respiratory rate ≥30 breaths/min, blood pressure (systolic <90 mm Hg, or diastolic ≤60 mm Hg) and age ≥65 years. AMS: Altered mental status; CAD: Coronary artery disease; CHF: Congestive heart failure; COPD: Chronic obstructive pulmonary disease; CURB-65: Confusion status; CVD: Cerebrovascular disease.

A total of 260 COVID-19 patients were admitted during the period between 27 February and 27 April 2020. COVID-19 testing for patients was done using RT-PCR through the Louisiana OPH Laboratory. The mean age was 58.6 ± 14.0 years (range: 18–93 years). Females represented 52.3% of the population, 86.2% were not Hispanic/Latino and 73.5% were African American; White patients accounted for 11.9% of the study population. BMI was 33.5 ± 8.7 kg/m^2^. One hundred seventy-nine (78.8%) had comorbid diseases. Hypertension (77.3%), obesity (59.6%), diabetes (41.9%) and malignancy (21.9%) were the most frequent. Clinical presentation and comorbidities are shown in Figure 1.

### Frequency of poor outcomes

Of the total COVID-19 patients, 131 patients (50.4%) required oxygen therapy, 70 patients (26.9%) required mechanical ventilation, 51 patients (19.6%) were intubated and 19 patients were on noninvasive ventilation (7.3%). During the hospital stay, 23 patients (8.8%) were extubated, nine of whom (3.5%) had unplanned reintubation. One hundred and four patients developed acute respiratory distress syndrome (ARDS), accounting for 40% of the study population. According to the degree of decrease in the oxygen content of the blood, the severity of ARDS was graded into mild, moderate and severe, representing 38.5%, 37.5% and 20.0% of patients, respectively. At the end of the study, 184 patients (70.8%) were discharged after a mean hospital stay of 9.64 ± 8.3 days, 36 patients (13.8%) were still hospitalized, and 40 cases (15.4%) died.

### Predictors for ICU admission & mortality in the study population

Seventy-six patients (29.2%) required ICU admission, 41 of whom (53.9%) were admitted directly to the ICU from the emergency department (ED). After adjustment of covariates, multivariable analysis showed that being obese (odds ratio [OR] = 4.46, 95% CI = 1.91–10.4, p = 0.001), an active smoker (OR = 2.43, 95% CI = 1.09–5.41, p = 0.028), having diabetes (OR = 2.17, 95% CI = 1.0–4.69, p = 0.048) or having high qSOFA score (OR = 2.46, 95% CI = 1.37–4.43, p = 0.003) indicated greater likelihood of being admitted to the ICU.

**Figure 2.**
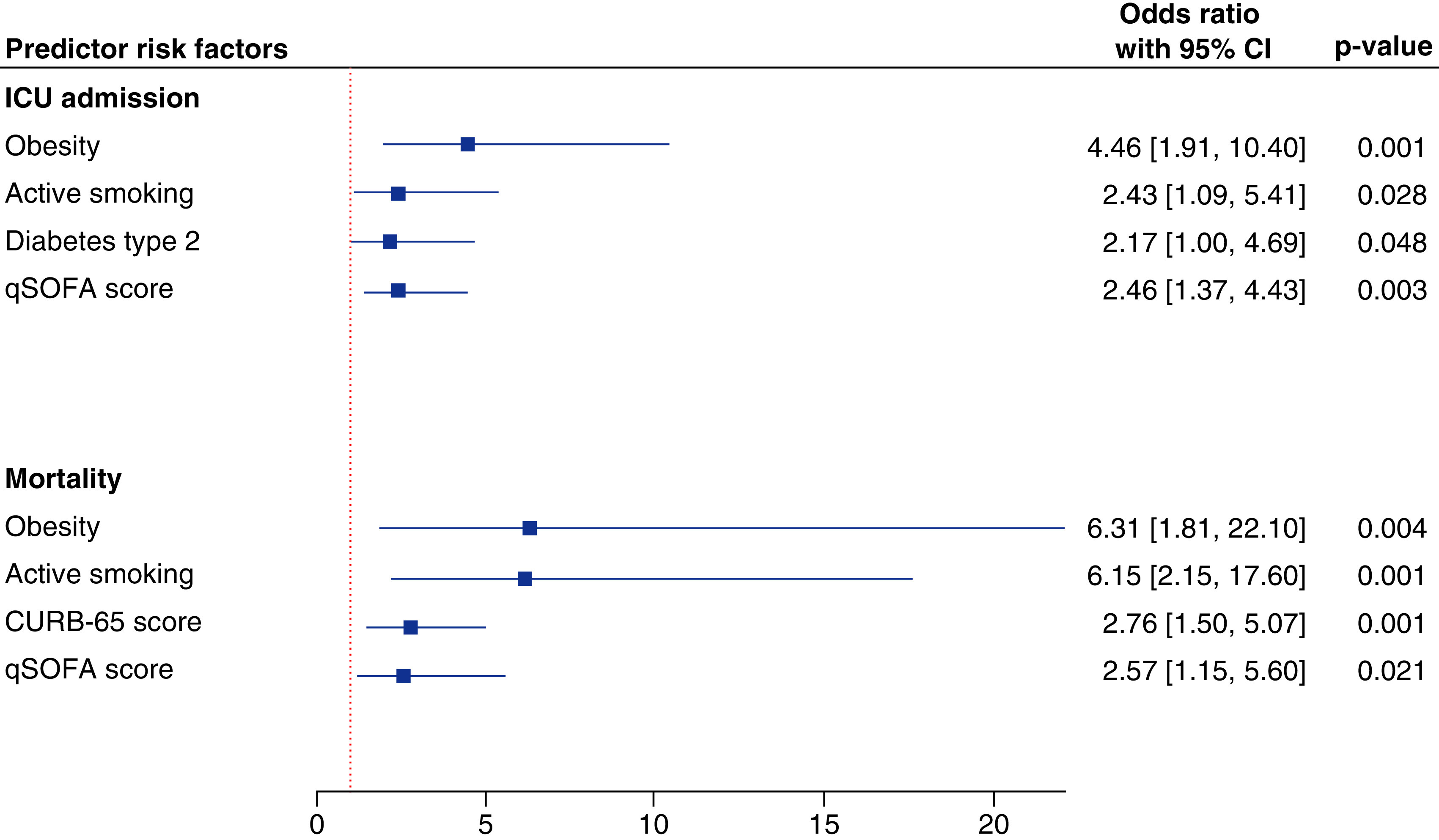
Independent predictor risk factors for ICU admission and mortality. Binary logistic regression analysis was performed, and data are reported as odds ratio and 95% CI. CURB-65, blood urea nitrogen level >19 mg/dl (>7 mmol/l), respiratory rate ≥30, blood pressure (systolic <90 mm Hg or diastolic ≤60 mm Hg) and age ≥65 years. CURB-65: Confusion status; ICU: Intensive care unit; qSOFA: Quick Sequential Organ Failure Assessment.

Mortality rate among patients admitted to the ICU was three times higher than those hospitalized in the general hospital floor (80.6 vs 19.4%, p < 0.001). In ward patients, 66.7% of deaths occurred within the first week, whereas in the ICU-admitted patients, higher death rate (60.7%) was remarkable in the second week. Most death in COVID-19 patients were attributed to ARDS (93.3%, p = 0.039). Regression analysis revealed that obesity (OR = 6.31, 95% CI = 1.81–22.1, p = 0.004), active smoking (OR = 6.15, 95% CI = 2.15–17.6, p = 0.001), CURB-65 score (OR = 2.76, 95% CI = 1.50–5.07, p = 0.001) and qSOFA score (OR = 2.57, 95% CI = 1.15–5.6, p = 0.021) were associated with an increased risk of mortality (Figure 2).

### Characteristics of cancer patients

**Figure 3.**
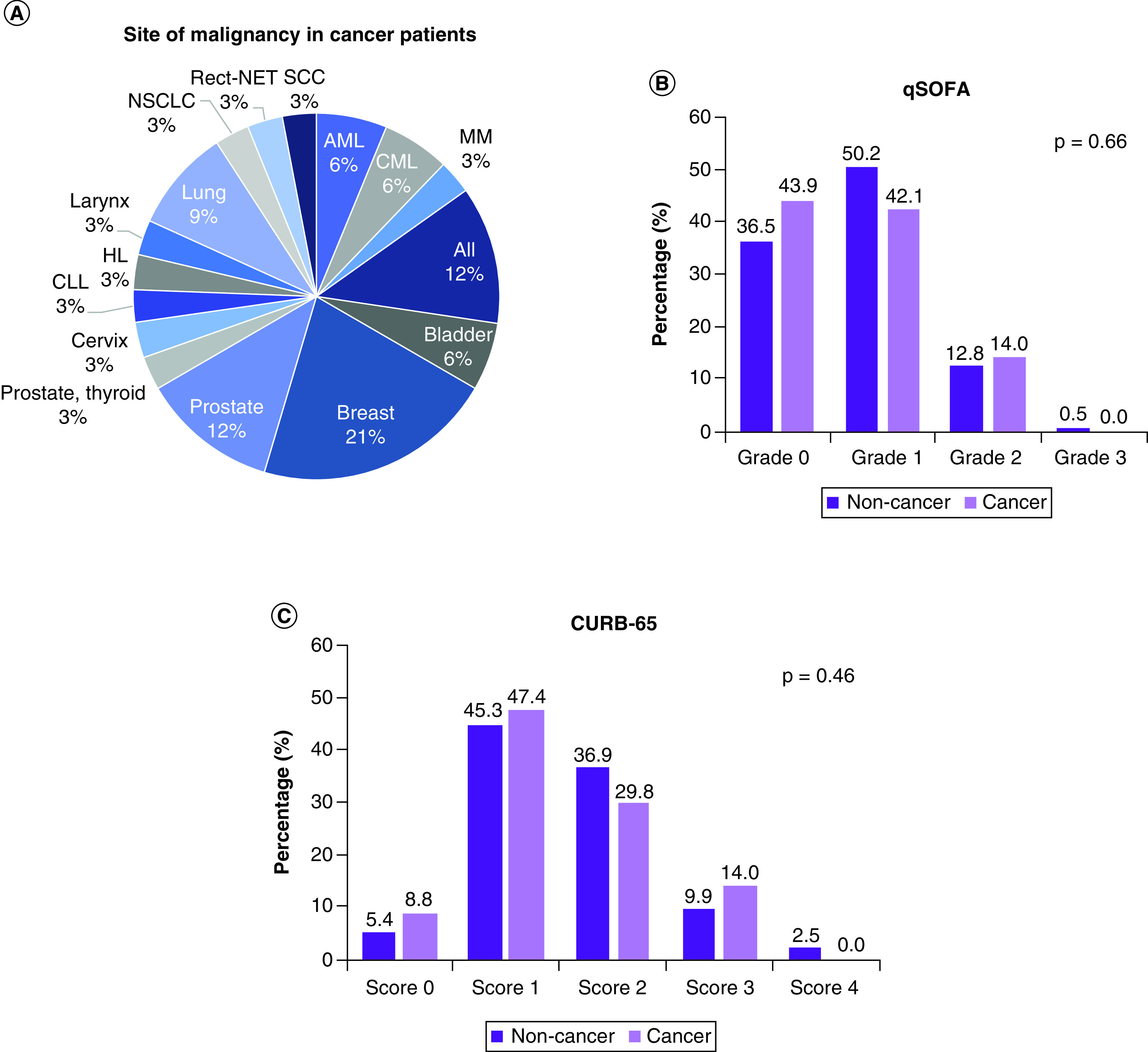
Characteristics of cancer and noncancer patients. **(A)** Frequency of tumor site in cancer group. **(B)** Frequency of cohorts according to qSOFA score. **(C)** Frequency of cohorts according to CURB-65 score. Chi-square test was used. CURB-65, blood urea nitrogen level >19 mg/dl (>7 mmol/l), respiratory rate ≥30, blood pressure (systolic <90 mm Hg or diastolic ≤60 mm Hg) and age ≥65 years. CURB-65: Confusion status; qSOFA: Quick Sequential Organ Failure Assessment.

Characteristics of 57 cancer (30 men and 27 women) and 203 noncancer patients were compared. Their mean age was 63.6 ± 12.5 years and 58.7 ± 14.6 years, respectively (p = 0.023). Breast and prostate cancers were the most common (Figure 3A). There was no significant difference in severity scores between cancer and noncancer cohorts (Figure 3B & C). As shown in Table 1, the prevalence of chronic kidney disease was double in individuals with cancer (26.3 vs 13.3%, p = 0.025), and chest pain and fatigue were more common in the cancer group (p = 0.006 and 0.020, respectively). Despite being older and having a higher frequency of comorbidities, there was no significant difference in ICU admission (22.2% vs 16.1%, p = 0.07), complications (78.8 vs 79.9%, p = 0.84) or mortality (12.3 vs 16.3%, p = 0.53). Analysis of COVID-19 patients who were discharged (n = 185) and those who were deceased (n = 40) is presented in Table 2. Of 49 closed cases, 42 cancer patients had been discharged accounting for a discharge rate of 85.7%. In contrast, 142 of 175 noncancer patients were discharged, representing 81.1%. Cohorts with cancer stayed longer in the hospital (12.8 ± 11.4 days) compared with noncancer patients (8.58 ± 6.50 days, p = 0.002).

**Table 1.** Comparison between cancer and noncancer patients.

Characteristics		Noncancer (n = 203)	Cancer (n = 57)	p-value
Age categories (yr)	<60	111 (54.7)	20 (35.1)	**0.011**
	≥60	92 (45.3)	37 (64.9)	
Gender	Female	109 (54.5)	27 (47.4)	0.37
	Male	91 (45.5)	30 (52.6)	
Ethnicity	Hispanic or Latino	2 (1.0)	1 (2.1)	0.36
	Not Hispanic or Latino	179 (91.8)	45 (95.7)	
	Not Reported	14 (7.2)	1 (2.1)	
Race	Black or African American	154 (77.0)	37 (66.1)	0.41
	White	23 (11.5)	8 (14.3)	
	Others	21 (10.5)	2 (3.6)	
Tobacco smoking	Never	127 (66.1)	30 (682)	0.40
	Current	44 (22.9)	12 (27.3)	
	Former	21 (10.9)	2 (4.5)	
Clinical presentation	SOB	118 (58.1)	27 (47.4)	0.17
	Cough	36 (17.7)	12 (21.1)	0.56
	Fever	38 (18.7)	11 (19.3)	0.92
	Chest pain	7 (3.4)	8 (14.0)	**0.006**
	Flu-like symptoms	11 (5.4)	4 (7.0)	0.74
	Fatigue	11 (5.4)	9 (15.8)	**0.020**
	Altered mental status	14 (6.9)	4 (7.0)	0.97
	Myalgia	2 (1.0)	3 (5.3)	0.07
	Seizure	3 (1.5)	0 (0.0)	0.35
	Headache	6 (3.0)	0 (0.0)	0.34
	Abdominal pain	3 (1.5)	3 (5.3)	0.12
	Bowel change	3 (1.5)	2 (3.5)	0.30
Comorbidities	Negative	77 (37.9)	4 (7.0)	**<0.001**
	Positive	126 (62.1)	53 (93.0)	
Type of comorbid disease	Hypertension	152 (74.9)	49 (86.0)	0.10
	Diabetes	84 (41.4)	25 (43.9)	0.76
	Obesity	125 (63.8)	30 (52.6)	0.16
	Cardiac disease	47 (23.2)	20 (35.1)	0.08
	CKD	27 (13.3)	15 (26.3)	**0.025**
	CVD	25 (12.3)	12 (21.1)	0.13
	COPD	21 (10.3)	5 (8.8)	0.72
	Asthma	27 (13.3)	4 (7.0)	0.25
ED disposition	Home	12 (6.7)	8 (14.8)	0.07
	Floor	139 (77.2)	34 (63.0)	
	ICU	29 (16.1)	12 (22.2)	
Current status	Still hospitalized	28 (13.8)	8 (14.0)	0.55
	Discharged	142 (70.0)	42 (85.7)	
	Deceased	33 (16.3)	7 (14.3)	
Complications	Negative	35 (20.1)	11 (21.2)	0.84
	Positive	139 (79.9)	41 (78.8)	
Mortality	Alive	170 (83.7)	50 (87.7)	0.53
	Dead	33 (16.3)	7 (12.3)	
Death location	Floor	8 (24.2)	2 (33.3)	0.63
	ICU	25 (75.8)	4 (66.7)	

Data are presented as number (percentage). Fisher's exact or chi-square tests were used.

CKD: Chronic kidney disease; COPD: Chronic obstructive pulmonary disease; CVD: Cerebrovascular disease; ED: Emergency department; ICU: intensive care unit.

**Table 2.** Impact of cancer on COVID-19 outcomes in closed cases.

Characteristics		Discharge		p-value	Deceased		p-value	Total		p-value
		Noncancer (n = 142)	Cancer (n = 42)		Noncancer (n = 33)	Cancer (n = 7)		Noncancer (n = 175)	Cancer (n = 49)	
Age	Mean ± SD	58.0 ± 15.1	63.8 ± 13.2	**0.026**	61.7 ± 11.3	64.0 ± 12.04	0.64	58.7 ± 14.6	63.6 ± 12.5	**0.023**
Gender	Female	80 (57.1)	20 (47.6)	0.29	15 (46.9)	3 (42.9)	0.84	95 (55.2)	23 (46.9)	0.30
	Male	60 (42.9)	22 (52.4)		17 (53.1)	4 (57.1)		77 (44.8)	26 (53.1)	
BMI	Mean ± SD	33.3 ± 9.08	31.35 ± 9.3	0.23	36.3 ± 7.66	33.2 ± 8.83	0.34	33.8 ± 8.74	32.1 ± 9.04	0.19
CURB-65	Mean ± SD	1.42 ± 0.73	1.31 ± 0.72	0.38	2.18 ± 1.01	2.00 ± 1.00	0.67	1.59 ± 0.84	1.49 ± 0.85	0.45
qSOFA	Mean ± SD	0.67 ± 0.60	0.67 ± 0.69	0.98	1.21 ± 0.82	1.00 ± 1.00	0.55	0.77 ± 0.68	0.70 ± 0.71	0.49
No. comorbidities	Mean ± SD	1.04 ± 1.25	2.29 ± 1.13	**<0.001**	1.24 ± 1.15	2.43 ± 1.51	**0.024**	1.12 ± 1.27	2.28 ± 1.22	**<0.001**
ABG	P/F ratio	242.7 ± 95	228 ± 137	0.65	139.8 ± 91.9	246.4 ± 118	**0.032**	208.4 ± 103	203 ± 127	0.83
Laboratory testing	CRP	36.0 ± 51.4	29.4 ± 40.4	0.60	35.7 ± 55.7	8.94 ± 5.67	0.30	33.0 ± 50.5	23.5 ± 33.7	0.34
	Ferritin	497 ± 423.7	717.5 ± 951	0.33	699.4 ± 439.3	515.8 ± 141.4	0.59	675.4 ± 689	901.4 ± 910	0.29
	Procalcitonin	16.6 ± 84.2	0.74 ± 1.37	0.60	60.5 ± 147.7	0.28 ± 0.29	0.52	19.5 ± 86.5	0.64 ± 1.04	0.40
	NLR	5.76 ± 3.91	9.32 ± 15.2	**0.016**	9.33 ± 7.42	5.08 ± 3.37	**0.029**	8.13 ± 22.3	8.81 ± 13.2	0.83
Require O_2_ therapy	Negative	60 (46.9)	19 (48.7)	0.85	13 (43.3)	2 (28.6)	0.67	73 (46.2)	21 (45.7)	0.94
	Positive	68 (53.1)	20 (51.3)		17 (56.7)	5 (71.4)		85 (53.8)	25 (54.3)	
Require intubation	Negative	113 (88.3)	34 (87.2)	0.78	7 (23.3)	3 (42.9)	0.36	120 (75.9)	37 (80.4)	0.69
	Positive	15 (11.7)	5 (12.8)		23 (76.7)	4 (57.1)		38 (24.1)	9 (19.6)	
Mechanical ventilation	Negative	100 (78.1)	30 (76.9)	0.83	6 (20)	4 (57.1)	0.06	106 (67.1)	34 (73.9)	0.47
	Positive	28 (21.9)	9 (23.1)		24 (80)	3 (42.9)		52 (32.9)	12 (26.1)	
Extubation	Negative	97 (91.5)	29 (87.9)	0.53	21 (75)	5 (71.4)	0.84	118 (88.1)	34 (85)	0.59
	Positive	9 (8.5)	4 (12.1)		7 (25)	2 (28.6)		16 (11.9)	6 (15)	
Unplanned reintubation	Negative	105 (99.1)	26 (92.9)	**0.049**	23 (82.1)	4 (80)	0.90	128 (95.5)	30 (90.9)	0.38
	Positive	1 (0.9)	2 (7.1)		5 (17.9)	1 (20)		6 (4.5)	3 (9.1)	
ICU admission	Floor	97 (77)	28 (71.8)	0.52	4 (13.3)	2 (28.6)	0.31	101 (64.7)	30 (65.2)	0.95
	ICU	29 (23)	11 (28.2)		26 (86.7)	5 (71.4)		55 (35.3)	16 (34.8)	
Develop complications	Negative	32 (22.9)	10 (24.4)	0.83	3 (9.4)	0 (0)	0.39	35 (20.3)	10 (20.8)	0.94
	Positive	108 (77.1)	31 (75.6)		29 (90.6)	7 (100)		137 (79.7)	38 (79.2)	
Type of complications	ARDS	26 (18.6)	9 (22)	0.65	14 (43.8)	3 (42.9)	0.96	40 (23.3)	12 (25)	0.84
	AKI	30 (21.4)	8 (19.5)	0.79	20 (62.5)	2 (28.6)	0.10	50 (29.1)	10 (20.8)	0.27
	Sepsis/shock	22 (15.7)	4 (9.8)	0.45	5 (15.6)	2 (28.6)	0.58	27 (15.7)	6 (12.5)	0.65
LOS	Total LOS	8.58 ± 6.50	12.8 ± 11.4	**0.002**	10.9 ± 6.48	16.5 ± 12.3	0.08	9.49 ± 6.39	14.4 ± 11.4	**<0.001**
	ICU LOS	1.09 ± 2.94	0.89 ± 2.63	0.71	6.88 ± 5.22	4.00 ± 4.65	0.19	2.42 ± 4.30	1.29 ± 3.09	0.09
	Ventilation	0.59 ± 2.27	0.84 ± 2.22	0.55	5.50 ± 4.09	3.00 ± 4.00	0.18	1.67 ± 3.44	1.06 ± 2.51	0.26

Data are presented as number (percentage) or mean ± SD. Fisher's exact or chi-square and Mann-Whitney or Student's t-tests were used, blood urea nitrogen level >19 mg/dl (>7 mmol/l), respiratory rate ≥30, blood pressure (systolic <90 mm Hg or diastolic ≤60 mm Hg) and age ≥65 years.

ABG: Arterial blood gases; AKI: Acute kidney injury; ARDS: Acute respiratory distress syndrome; CKD: Chronic kidney disease; COPD: Chronic obstructive pulmonary disease; CRP: C-reactive protein; CURB-65: Confusion status; CVD: Cerebrovascular disease; ICU: Intensive care unit; LOS: length of stay; NLR: Neutrophil-to-lymphocyte ratio; P/F ratio: PaO2/FiO2 ratio of arterial oxygen partial pressure (PaO2 in mmHg) to fractional inspired oxygen (FiO2 expressed as a fraction); qSOFA: Quick Sequential Organ Failure Assessment; SD: Standard deviation.

### Comparison between active & nonactive cancer patients

**Figure 4.**
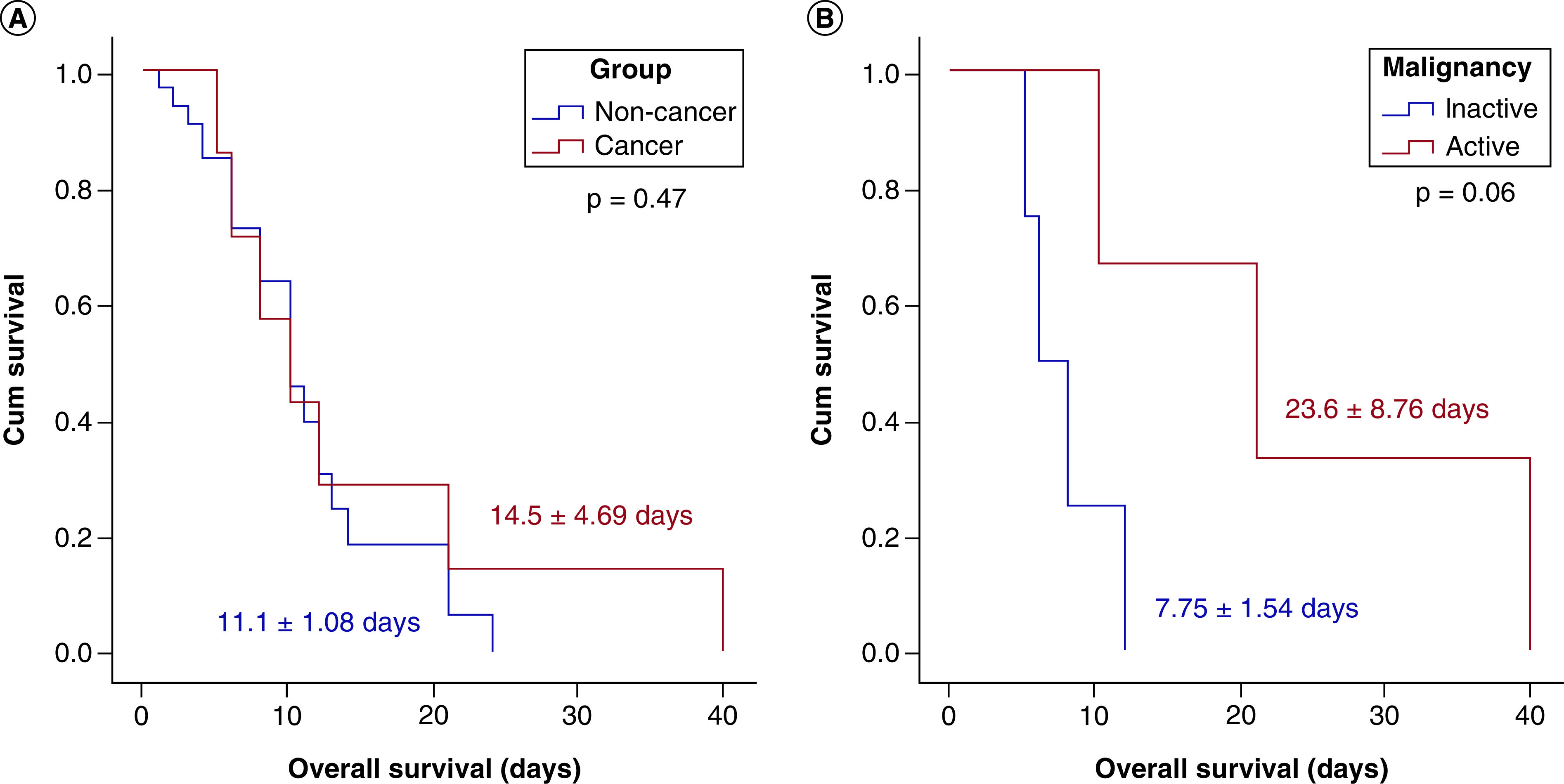
Kaplan-Meier survival curves. **(A)** Comparison between survival in cancer and noncancer groups. **(B)** Comparison between survival in active and nonactive cancer groups. Although the Kaplan-Meier curve shows different survival distribution between active and nonactive patients, it did not reach statistical significance (p = 0.06). Log rank test was used.

The comparison between active cancer patients with those who had a history of cancer showed no significant difference in their demographic and clinical features, laboratory data, complications or survival rate; however, active cancer cases required longer hospital stays (24.9 ± 17.1 vs 9.49 ± 6.39 days, p < 0.001) (Table 3). As depicted in Kaplan-Meier curves, there was no difference was observed in mortality between cancer and noncancer groups or between active and nonactive patients (Figure 4).

**Table 3.** Comparison between active and nonactive cancer patients.

Characteristics		Nonactive cancer (n = 46)	Active cancer (n = 11)	p-value
Demographics	Age	58.7 ± 14.6	60.7 ± 9.3	0.65
	Males	91 (45.5)	6 (54.5)	0.75
	BMI	33.8 ± 8.7	28.6 ± 7.8	0.05
Comorbidities	Diabetes	84 (41.4)	1 (9.1)	0.05
	Obesity	125 (63.8)	4 (36.4)	0.10
	HTN	152 (74.9)	9 (81.8)	0.60
	CAD	22 (10.8)	1 (9.1)	0.85
	CKD	27 (13.3)	2 (18.2)	0.64
	CURB-65	1.59 ± 0.8	1.27 ± 0.9	0.22
	qSOFA	0.77 ± 0.68	0.36 ± 0.50	0.05
	Number of comorbidities	1.12 ± 1.27	0.73 ± 0.6	0.30
ABG	P/F ratio	208.4 ± 103	261.8 ± 162.8	0.27
Laboratory testing	CRP	33.0 ± 50.5	12.2 ± 15.3	0.36
	Ferritin	675.4 ± 689	665.8 ± 628	0.98
	Procalcitonin	19.5 ± 86.5	0.09 ± 0.02	0.70
	NLR	8.13 ± 22.3	7.85 ± 7.63	0.96
Procedures	Require O_2_ therapy	100 (54.1)	6 (54.5)	0.97
	Require intubation	40 (21.6)	4 (36.4)	0.27
	Mechanical ventilation	56 (30.4)	3 (27.3)	0.82
	Extubation	16 (11.9)	3 (27.3)	0.15
	Unplanned reintubation	6 (4.4)	0 (0.0)	0.82
ICU admission	Floor	116 (66.7)	6 (54.5)	0.51
	ICU	58 (33.3)	5 (45.5)	
Complications	Positive	139 (79.9)	7 (63.6)	0.24
	ARDS	40 (23.0)	4 (36.4)	0.29
	AKI	50 (28.7)	2 (18.2)	0.73
	Sepsis/septic shock	28 (16.1)	1 (9.1)	0.53
Mortality	Positive	33 (16.3)	3 (27.3)	0.40
Death location	Floor	8 (24.2)	1 (33.3)	0.72
	ICU	25 (75.8)	2 (66.7)	
LOS	Total LOS	9.49 ± 6.39	24.9 ± 17.1	**<0.001**
	ICU LOS	2.41 ± 4.3	0.82 ± 1.94	0.22
	Ventilation	1.67 ± 3.4	0.82 ± 1.94	0.41

Data are presented as number (percentage) or mean ± SD. Fisher's exact or chi-square and Mann-Whitney or Student's t-tests were used, blood urea nitrogen level >19 mg/dl (>7 mmol/l), respiratory rate ≥30, blood pressure (systolic <90 mm Hg or diastolic ≤60 mm Hg) and age ≥65 years.

ABG: Arterial blood gases; AKI: Acute kidney injury; ARDS: Acute respiratory distress syndrome; CKD: Chronic kidney disease; COPD: Chronic obstructive pulmonary disease; CRP: C-reactive protein; CURB-65: Confusion status; CVD: Cerebrovascular disease; ICU: Intensive care unit; LOS: length of stay; NLR: Neutrophil-to-lymphocyte ratio; P/F ratio: PaO2/FiO2 ratio of arterial oxygen partial pressure (PaO2 in mmHg) to fractional inspired oxygen (FiO2 expressed as a fraction); qSOFA: Quick Sequential Organ Failure Assessment; SD: Standard deviation.

## Discussion

The COVID-19 pandemic has presented an incredible stress on the healthcare system. As of 5 September 2020, the total number of US COVID-19 infections had reached 6,095,007, with 185,687 deaths [15]. As the number of SARS-CoV-2 virus infections continues to rise, it has become increasingly important to characterize the disease process in varying patient populations for risk stratification.

One highly vulnerable group is cancer patients, who tend to be hospitalized for a prolonged period, increasing their risk of exposure to COVID-19 [16]. They have a weakened immune system due to the cancer itself and treatments such as chemotherapy or radiotherapy that they are undergoing. The current study included 260 COVID-19 patients in whom 57 cancer patients were compared with 203 noncancer patients, with breast and prostate cancer cases being the most frequent. Our findings indicate that cancer patients diagnosed with COVID-19 do not have significantly different outcomes in terms of adverse events or mortality rates than noncancer patients infected with COVID-19. Discharged cancer patients, however, did have higher rates of comorbidities, increased frequency of reintubation and prolonged length of hospital stay compared with noncancer COVID-19 patients.

In this study, cancer cohorts were older and more likely to have comorbid conditions. Similarly, different studies consistently reported that patients with a variety of comorbidities – including hypertension, diabetes and obesity – are at a higher risk of hospitalization due to COVID-19 than people without these conditions [17]. The data we compiled show a significantly higher number of comorbidities in cancer versus noncancer patients in both the deceased and discharged cohorts. In addition to underlying immunosuppression, the increased prevalence of comorbidities may be a contributing factor in the prolonged hospital stays seen in cancer patients relative to noncancer patients.

In our cancer cohorts, despite being older and having a higher frequency of comorbidities, there was no significant difference in ICU admission or risk of developing complications in cancer patients. However, it did show significantly longer hospital stays and higher rates of reintubation in the cancer cohort. This is consistent with the more severe disease course for cancer patients with COVID-19, as elucidated in previous studies [18–21]. It is also worth noting that active cancer patients experienced longer hospital stays than did either the nonactive cancer cohort or the noncancer group. Multivariable analysis, however, showed obesity, smoking and diabetes to be risk factors for unfavorable outcomes, consistent with prior studies [22,23].

While our data show a trend toward survival in nonactive patients, they do not demonstrate a significant difference in mortality rates between cancer and noncancer patients or between active and nonactive cancer groups. According to a study conducted in the UK on cancer patients receiving anticancer therapy, the mortality appeared to be driven by age, gender and comorbidities [24]. It was also reported that the COVID-19 mortality rate in breast cancer patients depends more on comorbidities than previous radiation therapy or current anticancer treatment [25].

Due to the recent pandemic, there was a delay of patient management with decrease in encounters in multiple fields (e.g., cardiology, breast, ophthalmology, oncology) [4]. It was suggested to postpone the screening and management of some cancer patients to avoid the risk of COVID-19 infection [26]. Also, Maringe *et al.* postulated that a considerable increase in the number of avoidable cancer mortality in England is to be expected due to diagnostic delays due to the COVID-19 pandemic in the UK [27]. However, Sundriyal *et al.* showed that modified oncological practice measures such as tele-consultations and providing supportive care at or near home and involvement of local/family physicians should be considered to minimize inpatient admissions; however, day-care chemotherapies were continued to provide optimal oncology services [28]. More research is required to identify the at-risk population and the management of cancer patients during the COVID-19 pandemic to avoid unnecessary delays in management and provide patients with optimal care because delaying management can lead to poor outcomes [29].

There are several potential limitations to this study. This cohort of COVID-19 patients is representative of the patient population at two university hospitals in New Orleans, Louisiana. A nationwide study involving several regions and a larger patient cohort may provide a more representative population from which to can identify the correlation between COVID-19 and cancer. Also, patients in New Orleans have varying frequencies of comorbidities compared with the rest of the country, which could potentially skew the data reflecting adverse outcomes and mortality. Lastly, our paper distinguished between active and nonactive cancer patients but made no distinction in the treatment modalities for patients with active malignancy. Future studies should seek to characterize active cancer patients by treatment modality to determine the effects that anticancer therapies have on COVID-19 disease progression as well as any alterations that should be made in treatment modalities for these patients.

## Conclusion

While cancer patients stayed longer in the hospital there was with no difference in mortality among cancer patients and the non cancer patients with the Worse outcomes in COVID-19 appear to be driven by obesity as well as active smoking.

This study will provide physicians and other healthcare workers valuable information regarding outcomes and anticipated clinical course for cancer patients diagnosed with COVID-19. As COVID-19 infection rates continue to remain high, it is our hope that this information will allow for better treatment and management of COVID-19 in patients with cancer.

**Summary points**

- Obesity, active smoking, diabetes and high Quick Sequential Organ Failure Assessment (qSOFA) score led to intensive care unit (ICU) admission.
- Mortality rates among patients admitted to the ICU were three times higher than patients in the ward.
- COVID-19 death was related mostly to acute respiratory distress syndrome.
- Obesity, active smoking, diabetes and high qSOFA and CURB-65 (Confusion status, blood urea nitrogen level >19 mg/dl [>7 mmol/l], respiratory rate ≥30, blood pressure [systolic <90 mm Hg or diastolic ≤60 mm Hg] and age ≥65 years) scores were associated with an increased risk of mortality.
- There was no significant difference in ICU admission between cancer and noncancer patients.
- There was no significant difference in the rate of complication between cancer and noncancer patients.
- Cohorts with cancer stayed longer in the hospital compared with noncancer patients.
- No difference was observed in mortality between cancer and noncancer groups or between active and nonactive patients.

## References

[1] CDC. CDC updates, expands list of people at risk of severe COVID-19 illness (2020). www.cdc.gov/media/releases/2020/p0625-update-expands-covid-19.html

[2] Clark A, Jit M, Warren-Gash C Global, regional, and national estimates of the population at increased risk of severe COVID-19 due to underlying health conditions in 2020: a modelling study. Lancet Glob. Health 8(8), e1003–e1017 (2020). 32553130 10.1016/S2214-109X(20)30264-3 PMC7295519

[3] Al-Tabba A, Al-Hussaini M, Mansour R Ethical considerations for treating cancer patients during the SARS-CoV-2 virus crisis: to treat or not to treat? A literature review and perspective from a cancer center in low-middle income country. Front. Med. (Lausanne) 7, 561168–561168 (2020). 33163499 10.3389/fmed.2020.561168 PMC7580805

[4] Toro MD, Brézin AP, Burdon M Early impact of COVID-19 outbreak on eye care: Insights from EUROCOVCAT group. Eur. J. Ophthalmol. 31(1), 5–9 (2020). 32967466 10.1177/1120672120960339

[5] Melidis C, Vantsos M. Ethical and practical considerations on cancer recommendations during COVID-19 pandemic [comment]. Mol. Clin. Oncol. 13(3), 5 (2020). 32754319 10.3892/mco.2020.2075 PMC7391840

[6] Liang W, Guan W, Chen R Cancer patients in SARS-CoV-2 infection: a nationwide analysis in China. Lancet Oncol. 21(3), 335–337 (2020). 32066541 10.1016/S1470-2045(20)30096-6 PMC7159000

[7] Wang H, Zhang L. Risk of COVID-19 for patients with cancer. Lancet Oncol. 21(4), e181 (2020). 32142621 10.1016/S1470-2045(20)30149-2 PMC7129735

[8] Desai A, Sachdeva S, Parekh T, Desai R. COVID-19 and cancer: lessons from a pooled meta-analysis. JCO Glob. Oncol. 6, 557–559 (2020). 32250659 10.1200/GO.20.00097 PMC7193801

[9] Newyork State Department of Health. COVID-19 Tracker (2020). https://covid19tracker.health.ny.gov/views/NYS-COVID19-Tracker/NYSDOHCOVID-19Tracker-Map

[10] Harris PA, Taylor R, Minor BL The REDCap consortium: Building an international community of software platform partners. J. Biomed. Inform. 95, 103208 (2019). 31078660 10.1016/j.jbi.2019.103208 PMC7254481

[11] Kao HC, Lai TY, Hung HL Sequential oxygenation index and organ dysfunction assessment within the first 3 days of mechanical ventilation predict the outcome of adult patients with severe acute respiratory failure. ScientificWorldJournal 2013, 413216 (2013). 23476133 10.1155/2013/413216 PMC3588184

[12] Yang AP, Liu JP, Tao WQ, Li HM. The diagnostic and predictive role of NLR, d-NLR and PLR in COVID-19 patients. Int. Immunopharmacol. 84, 106504 (2020). 32304994 10.1016/j.intimp.2020.106504 PMC7152924

[13] Su Y, Tu GW, Ju MJ Comparison of CRB-65 and quick sepsis-related organ failure assessment for predicting the need for intensive respiratory or vasopressor support in patients with COVID-19. J. Infec. 81(4), 647–679 (2020). 10.1016/j.jinf.2020.05.007 PMC7204730 32389785

[14] George N, Elie-Turenne MC, Seethala RR External validation of the qSOFA score in emergency department patients with pneumonia. J. Emerg. Med. 57(6), 755–764 (2019). 31735660 10.1016/j.jemermed.2019.08.043

[15] WHO. WHO Coronavirus Disease (COVID-19) Dashboard, United States of America (2020). https://covid19.who.int/region/amro/country/us

[16] CDC. CDC updates, expands list of people at risk of severe COVID-19 illness (2020). www.cdc.gov/media/releases/2020/p0625-update-expands-covid-19.html

[17] CDC. COVID-19-associated hospitalization related to underlying medical conditions (2020). www.cdc.gov/coronavirus/2019-ncov/covid-data/investigations-discovery/hospitalization-underlying-medical-conditions.html

[18] Liang W, Guan W, Chen R Cancer patients in SARS-CoV-2 infection: a nationwide analysis in China. Lancet Oncol. 21(3), 335–337 (2020). 32066541 10.1016/S1470-2045(20)30096-6 PMC7159000

[19] Dai M, Liu D, Liu M Patients with cancer appear more vulnerable to SARS-CoV-2: a multicenter study during the COVID-19 outbreak. 10(6), 783–791 (2020). 10.1158/2159-8290.CD-20-0422 PMC7309152 32345594

[20] Gosain R, Abdou Y, Singh A COVID-19 and Cancer: a comprehensive review. Curr. Oncol. Rep. 22(5), 53 (2020). 32385672 10.1007/s11912-020-00934-7 PMC7206576

[21] Ueda M, Martins R, Hendrie PC Managing cancer care during the COVID-19 pandemic: agility and collaboration toward a common goal. J. Natl Compr. Canc. Netw. 1–4 (2020) (Epub ahead of print). 10.6004/jnccn.2020.7560 32197238

[22] Richardson S, Hirsch JS, Narasimhan M Presenting characteristics, comorbidities, and outcomes among 5700 patients hospitalized with COVID-19 in the New York City Area. JAMA 323(20), 2052–2059 (2020). 32320003 10.1001/jama.2020.6775 PMC7177629

[23] Sanyaolu A, Okorie C, Marinkovic A Comorbidity and its impact on patients with COVID-19. SN Compr. Clin. Med. 1–8 (2020) (Epub ahead of print). 10.1007/s42399-020-00363-4 PMC7314621 32838147

[24] Lee LYW, Cazier J-B, Angelis V COVID-19 mortality in patients with cancer on chemotherapy or other anticancer treatments: a prospective cohort study. Lancet 395(10241), 1919–1926 (2020). 32473682 10.1016/S0140-6736(20)31173-9 PMC7255715

[25] Vuagnat P, Frelaut M, Ramtohul T COVID-19 in breast cancer patients: a cohort at the Institut Curie hospitals in the Paris area. Breast Cancer Res. 22(1), 55 (2020). 32460829 10.1186/s13058-020-01293-8 PMC7254663

[26] ESMO. Cancer patient management during the COVID-19 pandemic (2020). www.esmo.org/guidelines/cancer-patient-management-during-the-covid-19-pandemic

[27] Maringe C, Spicer J, Morris M The impact of the COVID-19 pandemic on cancer deaths due to delays in diagnosis in England, UK: a national, population-based, modelling study. Lancet Oncol. 21(8), 1023–1034 (2020). 32702310 10.1016/S1470-2045(20)30388-0 PMC7417808

[28] Sundriyal D, Sehrawat A, Kumar P, Bhandari R. Impact of COVID-19 pandemic on oncology practices during nationwide lockdown period: a single centre experience and the way forward. J. Assoc. Physicians India 68(7), 48–50 (2020). 32602680

[29] Elfalah M, Alryalat SA, Toro MD Delayed intravitreal anti-VEGF therapy for patients during the COVID-19 lockdown: an ethical endeavor. Clin. Ophthalmol. 15, 661–669 (2021). 33628009 10.2147/OPTH.S289068 PMC7898208

